# Exacerbating Inequalities? Health Policy and the Behavioural Sciences

**DOI:** 10.1007/s10728-018-0357-y

**Published:** 2018-04-11

**Authors:** Kathryn MacKay, Muireann Quigley

**Affiliations:** 1 Politics, Philosophy, and Religion Department, Lancaster University, Lancaster, UK; 2 Birmingham Law School, University of Birmingham, Birmingham, UK

**Keywords:** Inequity, Public health, Social determinants, Behavioural science, Health policy, Regulation

## Abstract

There have been calls for some time for a new approach to public health in the United Kingdom and beyond. This is consequent on the recognition and acceptance that health problems often have a complex and multi-faceted aetiology. At the same time, policies which utilise insights from research in behavioural economics and psychology (‘behavioural science’) have gained prominence on the political agenda. The relationship between the social determinants of health (SDoH) and behavioural science in health policy has not hitherto been explored. Given the on-going presence of strategies based on findings from behavioural science in policy-making on the political agenda, an examination of this is warranted. This paper begins by looking at the place of the SDoH within public health, before outlining, in brief, the recent drive towards utilising behavioural science to formulate law and public policy. We then examine the relationship between this and the SDoH. We argue that behavioural public health policy is, to a certain extent, blind to the social and other determinants of health. In section three, we examine ways in which such policies may perpetuate and/or exacerbate health inequities and social injustices. We argue that problems in this respect may be compounded by assumptions and practices which are built into some behavioural science methodologies. We also argue that incremental individual gains may not be enough. As such, population-level measures are sometimes necessary. In section four we defend this contention, arguing that an equitable and justifiable public health requires such measures.

## Introduction

There have been calls for some time for a new approach to public health in the United Kingdom and beyond. This is consequent on the recognition and acceptance that health problems often have a complex and multi-faceted aetiology [42, 74]. In particular, there is evidence that a range of social and other factors influence individual health outcomes. These include early health status, educational attainment, employment (or lack thereof), working conditions, and income level [42]. This has, in turn, been acknowledged in a variety of Government reports [23, 35, 42]. At the same time, policies which utilise insights from research in behavioural economics and psychology (henceforth ‘behavioural science’^1^) have gained prominence on the political agenda, including those insights which purport to help people make decisions which are better for their health. This research broadly concludes that we make certain predictable and systematic errors in judgement. Our choices and decisions are affected by our reliance upon a range of cognitive biases and heuristics. For instance, some research shows that the way information is presented to us influences the way we choose (framing effect), we tend to show undue confidence regarding the probability that events will or will not occur (optimism bias), and we do not tend to deviate from default options which are presented to us (*status quo* bias).^2^ The principal message to be taken from behavioural science research is that contextual influences (sometimes called choice architecture [73]) affect the decisions we make. By taking account of these, government and regulators can thus attempt to alter the health behaviour of citizens through approaches which harness or eliminate our cognitive quirks.

The relationship between the social determinants of health (SDoH) and behavioural science in health policy has not hitherto been explored. Given the on-going presence of strategies based on findings from behavioural science in policy-making on the political agenda (nationally and internationally [18]), an examination of this is warranted. Making explicit the connections or (as it turns out) lack thereof between these two areas highlights potential problems with the use of the behavioural sciences in public health policy. To this end, we begin in the next section by looking at the place of the SDoH within public health, before outlining, in brief, the recent drive towards utilising behavioural science to formulate law and public policy. We then examine the relationship between this and the SDoH. Our argument here is that new behavioural public health policy is, to a certain extent, blind to the social and other determinants of health. Suggested and actual applications of the behavioural sciences in health policy have mainly targeted a group of issues that have become the usual public health suspects (smoking, diet, alcohol intake, and physical activity). This, we suggest, is problematic. Without adequate consideration of the wider social and other contexts of health, this could have important equity and justice implications. Thus, in “(In)equality and Social (In)justice I: Methodological and Empirical Concerns” section, we examine ways in which such policies may perpetuate and/or exacerbate health inequities and social injustices. We argue that problems in this respect may be compounded by assumptions and practices which are built into some behavioural science methodologies. Here we also argue that the incremental individual gains, which some behavioural science interventions bring about, may not be enough. As such, population-level measures are sometimes necessary. In “(In)equality and Social (In)justice II: What Kind of public Health?” section we defend this contention, arguing that an equitable and justifiable public health requires such measures.

## SDoH and the Behavioural Sciences

### Two Approaches to Health

Between the 1970s and 1990s, researchers began examining the ‘causes of the causes’ of poor health. They were interested in the social conditions that give rise to a higher risk of non-communicable disease in some groups, and studied the differences in mortality rates in British civil servants. In particular, Marmot and Rose’s Whitehall studies provided evidence that the relationship between poverty and health may not be characterised by a threshold, but by a gradient. It is not the case, they found, that achieving a certain socio-economic status is *sufficient* to achieve good health. Instead, throughout class ranks each group does better in health than the one below [50, 51]. Marmot and Rose theorised that these disparities in health were caused by a combination of unhealthy behaviours and the effects of impossibly stressful lives [42, 51, 69]. The ‘social determinants’, as they became known, include factors like housing and living environment, exposure to environmental pollutants, educational attainment, food quality and availability, and many other factors that impact our health, outside of genetic predispositions or accidental illness or injury. Since the 1990s, a growing amount of research has confirmed the observation that those with greater economic and social resources are more likely to have better health outcomes insofar as they may do better regarding the social determinants, resulting in predictable disparities in health between groups [13, 19, 32, 41, 63].

Despite this research, establishing causation between socio-economic status and health outcomes has been difficult because of the various complex, and often covert, ways in which determinants like housing, employment, transportation, educational attainment, childcare, food provision, and a variety of other factors have a cumulative impact upon health over time. As such, teasing out and analysing the multiplicity of covariates has proved challenging. However, researchers continue to find correlations between poor health and low socioeconomic status. For example, a recent analysis of active transportation among school-age children in California found an unexpected positive relation between using active transit to get to school and having obesity. The researchers proposed that the link between these factors may be poverty: children who are poor are both more likely to bike or walk to school and also to be obese [17]. As such, public health’s messaging to groups with higher rates of obesity to be more active may miss the audience, and the point. Other researchers in the US have found that racial and economic disparities lead to worse health outcomes and higher obesity rates [2, 17, 79]. Some commentators have noted that results like these reveal a blind spot in the public health policy literature. While public health researchers seem to have reached a consensus that low socioeconomic status begets poor health, many of the policy reports in public health continue to call for individual changes, not the mitigation of social determinants connected to poverty, or the regulation of various social systems (such as the food provision system) [17, 68].

Amongst the reports which emphasise individual changes are those which propose policy influenced by behavioural science. Although behaviour-change approaches are not new, behavioural science-influenced strategies have come to the political fore in recent times. The Behavioural Insights Team (BIT) in the UK, established in 2010, leads the way in this regard. Sometimes called the ‘nudge unit’, BIT originally started within the Cabinet Office, before separating out and becoming a public–private partnership with both the Cabinet Office and the National Endowment for Science, Technology, and the Arts [7]. The influence of the UK-based team spread internationally. Members of the BIT went on to advise the New South Wales Office of Premier and Cabinet in Australia on a range of policy measures. That Office now has its own team [24]. Following this, in 2015 the Australian Government created a Federal level unit [25]. In the US, under the Obama administration, the White House set up the Social and Behavioural Sciences Team (SBST). This team was created with the aim of translating “findings and methods from the social and behavioral sciences into improvements in Federal policies and programs” [71], which was subsequently specified in an Executive order [56]. The fate of the SBST under the current administration is yet uncertain (but appears safe). Across Europe, several countries also have dedicated behavioural teams within Government or are explicitly using behavioural science research to inform policy-making; for instance, the Netherlands, Germany, and Denmark [28]. Moreover, at European Commission level, the Joint Research Centre (JRC) provides support for behavioural research in Commission service areas [27].

Both the BIT and the European Commission’s JRC have produced reports which encompass a range of policy areas, including the application of behavioural science to health [4, 5, 37, 76]. To highlight the kinds of interventions being trialed and implemented, and the findings from behavioural science which inform them, consider some measures in relation to smoking. In the UK and Iceland, it is illegal to openly display tobacco products, and in the UK, Ireland and France, plain packaging regulations are in force. In the UK, this legislation came into force in May 2017, following an unsuccessful legal challenge by the tobacco industry [26]. Draft plain packaging regulations are also being considered in a number of other European countries [28]. Both restrictions on display and plain packaging initiatives aim to decrease the visibility and social acceptability of tobacco products. The way these measures work is two-fold. First, the visual cues which can trigger consumption are reduced by restricting displays. Second, the power of social norms is harnessed. By hiding the products and removing ‘attractive’ and branded packaging, these measures send a message that smoking is not socially acceptable. Although these initiatives involve legislation, the behaviour of individual citizens is not being *directly* regulated. Whilst it may be illegal for shops to display tobacco products, it is not illegal to smoke, and the committed smoker can still buy cigarettes. There is no ban on tobacco products, or their purchase or sale. It is for this reason that strategies such as these, which alter the choice environment, are sometimes labelled ‘nudges’ or ‘libertarian paternalism’ [59, 73].

Consider also food and diet. In a recent BIT report on health, suggestions relating to food include decreasing the size of food packaging and tableware, and changing the placement of healthy foods in shops to make them more accessible (conversely one could make ‘unhealthy’ foods less accessible) [73]. Such suggestions draw on research which points to the biases and other factors which affect eating. For instance, ‘present bias’ means that we give more weight to our present desires, and tend to discount our future or longer-term goals. This can manifest through undervaluing the longer-term disadvantages of unhealthy diet or of skipping exercise, and overvaluing the immediate benefits of food experiences. The benefits of hyper-palatable food (food that is created to be very high in salt, sugar, and fat) to us *now* is more salient than any longer-term disadvantages it may have. As such, we might favour high-convenience food in the present, seeking to impose greater self-control on our future selves. We tell ourselves ‘I’ll eat better tomorrow’, something which may not materialise [46]. In addition to present bias, things like menu layout in restaurants, as well as portion sizes can affect what we choose and how much we eat [46]. Rather than placing regulations around the added ingredients in hyper-palatable food, policies informed by behavioural science often suggest changing the decision environment; for example, calorie-postings on menu boards, changing the size of plates at self-serve food outlets, or rearranging food displays.^3^ These are individual-focused initiatives, which operate on a one-person-at-a-time basis, and thus, the success of these measures to change eating behaviours are highly variable between people. In “(In)equality and Social (In)justice II: What Kind of public Health?” section, we will discuss a regulation in the food system which is not individual-focused, and which has great public health potential.

### Ships in the Night?

The drive to utilise behavioural science in (public) health policy has thus far taken little account of macro-level social determinants. Take, for example, three prominent policy reports: (1) the 2010 Behavioural Insights Team’s *Applying Behavioural Insight to Health* [4]; (2) the European Commission Joint Research Centre’s (JRC) 2013 ‘Applying Behavioural Sciences to EU Policy-making’ [75]; and (3) the JRC’s most recent ‘Behavioural Insights Applied to Policy European Report 2016’ [47]. The BIT report is exclusively focused on health. The other two are more broadly focused, but they mention health and health-related interventions to varying degrees. As can be seen from examples given in the previous section, the usual public health suspects are a prevalent focus in policy reports drawing on research in behavioural science. Smoking, alcohol, diet, and physical activity take up the greater part of health issues discussed across all three reports. The 2010 BIT report discusses other topics, examining organ donation, teenage pregnancy, diabetes control and compliance, and food hygiene [4]. Nevertheless, we can see that this list does not include the ‘social, material, political and cultural inequalities’ which constitute the macro determinants of health status and outcomes. The 2016 BIT report on health reaffirms this ‘lifestyle’ focus, saying:

… Around half of the global burden of disease arises from behavioral and lifestyle factors. Unhealthy eating, smoking and alcohol consumption contribute to the development of long-term conditions such as diabetes; cardiovascular diseases; chronic respiratory diseases; and musculoskeletal disorders [37].

Yet, despite the report noting that we need to better understand the “ways that ill-health develops” and “why unhealthy behaviours happen” [37], it contains no engagement with issues regarding the social determinants of health.

The academic literature does not fare much better. The two areas—behavioural science and the social determinants of health—currently occupy two almost separate spheres of academic debate. While some articles mention these together, there is not generally any substantive discussion of the relationship (or potential relationship) between them.^4^ From the behavioural sciences side of things, some articles include references to social determinants, while not actually being about them. For example, Glanz and Bishop state in the abstract to their paper that “influential contemporary perspectives stress the multiple determinants and multiple levels of determinants of health and health behaviour” [33]. They write that public health and health promotion initiatives will be most effective if they are based on an ecological understanding of health behaviours, which are attentive to the social and political environment a person has to operate within. However, it becomes clear that Glanz and Bishop, like other commentators, find choice architecture to be a sufficiently ecological approach. They provide an overview of the theories of behavioural science as they apply to health promotion, and then claim that the most prominent contributors to death and disease in the US and globally are individual behavioural factors [33]. Thus, while their piece initially appears to engage with the SDoH, the focus on behavioural factors and choice architecture actually leads to their exclusion [33, 44, 68]. Others, such as Roberto and Kawachi, fare slightly better, offering a brief consideration of how behavioural strategies could complement other “long-term and structural barriers to achieving optimal health in people’s lives” [64]. Here there is a broad acknowledgement that wider factors are determinative of a person’s health. But again there is no in-depth engagement with this and with the (potential) relationship with behavioural science-inspired approaches.

Those articles and documents which approach the issue from a public health or SDoH perspective engage with behavioural science in a more substantive fashion than vice versa. However, this engagement is critical in nature. The criticism of behavioural science from within public health is exemplified by a recent WHO report that suggests that a focus on individual behaviours and nudges contribute to the failure to address health equity and the social determinants of health in Europe. The report noted that WHO studies found a tendency “to focus on intermediate or proximal determinants such as access to health services, lifestyle or behaviour, living conditions (housing, water and sanitation) and social cohesion” [80]. This focus was symptomatic of a failure to properly conceptualise the multiple factors involved in the health of a population, and to “intervene with the magnitude and intensity necessary to affect their distribution” [80]. The proposed reasons for this echo the Marmot Working Committee’s findings outlined earlier; that is, the tendency to focus on proximal issues affecting health is influenced by many factors, including political ideology and the interests of different stakeholders^5^ [80]. The WHO report notes that part of this “includes a resurgence of the trickle-down effect and a focus on individual responsibilities and behaviour change, such as ‘nudge’ strategies” [80]. Taken in the broader context of the report, this represents a clear statement that the WHO does not think that nudge-type interventions from behavioural science can address significant health concerns, health equity, or the fundamental social determinants of health.

Similar critiques can also be found in other places. For instance, Rayner and Lang argue that behaviourally-focused policies in their current form do not take account of the complicated reality of the decisions that most people face [61]. There is also a concern that nudges provide at best a smokescreen for governmental inaction, and at worst a marketing ploy in concert with industry actors [11, 48, 53, 61]. Regarding social determinants, Bonell and colleagues argue that the UK government’s promotion of nudging as an alternative to ‘hard’ policy interventions misrepresents the original theory behind nudging (as one of a suite of regulatory tools). Misleading the public about the nature of nudges “serves to obscure the government’s failure to propose realistic actions to address the upstream socioeconomic and environmental determinants of disease” [11]. A related, but deeper, issue with the emphasis on nudge-type initiatives is that public health is reduced to the ‘managerial’ [45]. Lang and Rayner surmise that the increasingly technical language of policy ‘delivery’, and now the micro-level focus on nudging individuals, frames thought around health and well-being in such a way that it discourages public health attention on the “macro, big picture, framing contexts of life” [45]. Nudge, for them, is the current fad within the general trend towards a managerial and reductionist approach to public health, which ultimately threatens to make public health irrelevant [45].

## (In)equality and Social (In)justice I: Methodological and Empirical Concerns

As we have just seen, the behavioural science and social determinants of health literatures are to a large extent disconnected. This is concerning because, by not taking account of the social and other macro determinants of health, policies which draw on the behavioural sciences will continue to have an overly narrow and individualistic focus. They will fail to make any real headway in tackling the root causes of ill-health and health inequalities. There are a number of methodological and empirical concerns regarding the behavioural sciences which have the potential to compound this problem. While policy-oriented behavioural scientists seem aware of some of the limitations of their research [66], and have recently outlined a number of issues regarding the replicability or generalisability of their findings, concerns remain which need to be addressed if behavioural science- inspired policy is to make any real headway in public health. The first of these concerns the evidence-base for the use of behavioural insights in public health law and policy. The second concerns the effectiveness of potential behavioural science interventions in the public health arena in general.

### Behavioural Law and Policy: Problems of Translation

Translating behavioural sciences research into law and policy is not necessarily straightforward. In particular, there are empirical and methodological limits which may lead to existing inequalities being perpetuated or exacerbated. One difficulty is that much initial research in the behavioural sciences is carried out in laboratory-like conditions on populations which have been described as **WEIRD**; that is, it is conducted on research participants who are **W**estern, **E**ducated, and from **I**ndustrialized, **R**ich, and **D**emocratic countries [40]. The difficulty with this is that such groups are commonly treated *as if* they are representative of populations more generally or the intended target group for a real-world intervention. This, however, ought not to be assumed. Henrich and colleagues conducted a review of large-scale studies which involved comparative experiments on behavioural variables. In this review, they made four comparisons: (1) industrialised societies versus small-scale societies; (2) western versus non-western societies; (3) contemporary Americans versus the rest of the West; (4) typical contemporary American subjects versus other Americans. They concluded that, rather than WEIRD research participants being representative, they are outliers across multiple dimensions. Consider just two findings. In comparing differences within Western populations, they found that Americans have a more individualistic self-concept that other Western countries, valuing independence and choice to a higher degree [40]. And in making comparisons between different types of American subjects, they found that undergraduate student test-subjects displayed more prosocial tendencies than older adults. They displayed higher levels of “trust, fairness, cooperation, and punishment of unfairness or free-riding” in experimental measures of these [40].

Hence, whilst a substantial portion of behavioural studies involve WEIRD populations, these are biased population samples. As Henrich and colleagues put it:

Sampling from a thin slice of humanity would be less problematic if researchers confined their interpretations to the populations from which they sampled. However, despite their narrow samples, behavioral scientists often are interested in drawing inferences about the human mind and human behavior. This inferential step is rarely challenged or defended… despite the lack of any general effort to assess how well results from WEIRD samples generalize to the species [40].

In short, biased samples produce biased data. When this happens, we risk getting non-representative, non-diverse, non-generalisable results, something which is even more problematic when imported into law and policy. But, even if WEIRD populations were representative of the cognitive biases displayed by populations generally (e.g. the UK population), this is not a guarantee that they are representative of *particular* target populations for *particular* interventions (e.g. women aged 30–40, who are living in poverty in the North East of England). Moreover, some of what is termed ‘cognitive bias’ may have social and cultural elements [67]. The consequence of all this might be that the desired effect of the behavioural science-inspired policy may not work in practice when transplanted to different populations. This is something which may be particularly significant given the heterogeneous demographics amongst, for instance, EU member states, economically, socially, politically, and so on^6^ [60].

Leaving the issue of WEIRD populations aside, there are other problems with the use of these laboratory-type experiments. Much of the relevant research is done in controlled laboratory-like settings, and can include ‘game play’ research methods (e.g. ultimatum bargaining, public goods games, and prisoner’s dilemmas) where there are no real-life costs or benefits associated with them [8, 9, 12, 15]. These experiments are designed with specific parameters and controls. Therefore, they may not capture the complexity of the real-world environment or the day-to-day reality of human behaviour.

The difficulties with the experimental studies are compounded by the fact that there is a lack of robust empirical evidence from applied settings that can point to whether or not the findings of such research translate well outside of the experimental context. One solution to this, which the BIT advocates, is the use of RCTs. These studies involve testing interventions by randomly allocating test subjects to the interventions. There is also a control group which does not receive an intervention (or receives the currently best available one for comparison). Conducting RCTs certainly represents an improvement over not doing any applied studies for behavioural interventions. However, there are at least three difficulties with these. First, we do not yet have many studies of this kind which test public health-related interventions. Second, even where such studies have been conducted, these have been over a short timescale. As such, there are questions about the long-term effects of interventions within complex real-work settings. As members of the BIT have recently noted, “[o]ne underlying issue is that public officials and academics (particularly junior scholars) are rarely incentivised to choose studies where the main outcome measure will only be reported far in the future” [66]. Third, there are questions of applicability. In a similar to manner to experimental studies, the results of RCTs conducted in a particular place under particular conditions do not necessarily or automatically apply to other places with different conditions [16, 22, 30].

Finally, of those RCTs that have been conducted, the persuasiveness of the evidence is sometimes questionable. Take, for instance, an RCT on smoking cessation conducted by the BIT for Public Health England (PHE). The BIT tested the use of different messages and images on a website aimed at encouraging people to stop smoking [6]. According to a PHE report, this trial “found that website design and time of visit affected registrations by up to 5.9 percentage points and the optimal registration page included no picture, a testimonial and a health benefits message” [57]. Moreover, the trial resulted in 2040 more registrations to the smoking cessation programme [57]. The problem with this is twofold. First, an interrogation of the graph of the different interventions trialled shows that the actual difference between the *control* and the most effective intervention looks to be less than one percentage point. This does not appear to be particularly efficacious. Second, the measured outcome was *registrations* to the smoking cessation programme, not actual smoking cessation. Therefore, if just over 2000 people registered, then we can expect significantly fewer to have stuck with the programme and quit. Again this does not seem to be particularly effective, especially given that 345,920 webpage landings were recorded during the trial.

In sum, the supposed evidence behind proposed behavioural science-informed health law and policy initiatives is at times limited both in terms of how much evidence supports the research findings, and in terms of how well the findings apply to new contexts.

### Individual Incremental Gains or Across the Board Regulation?

One response to the above critique is to say that some health benefits are better than none, and that we ought to use behavioural science in law and policy where such gains can be made. So, in relation to the smoking trial, we might say that at least some people will have stopped smoking and this is a good thing in relation to harm reduction and their health. Indeed, some version of this argument seems to be at work for proponents of these new behavioural science-informed approaches. For example, Hallsworth contends that public health measures should focus on interventions which can give progressive marginal gains and harness our more automatic, habitual behaviours [38]. We do not want to suggest that marginal gains might not lead to some change, especially when compounded over time. However, individual incremental gains are highly inconsistent—as uptake varies between individuals—and may not be enough to effect large-scale change, either quickly enough or in a cost-effective manner. They also may not be taken up by the individuals most in need of public health interventions [3]. More may be needed, such as regulation on upstream health factors. To illustrate, let us consider two such examples: trans fats and sugar.

Trans fats are a food additive that industry has been using for over a century to lengthen the shelf-life, improve palatability, and stabilise for deep-frying a number of processed foods [14]. These fats are also closely associated with increases in the incidence of high levels of ‘bad’ cholesterol (low-density lipoprotein) in the blood, and increased risk of cardiovascular disease [14, 62]. There is accumulating evidence that people with less money, less education, insecure employment and poor housing conditions are more likely to experience food insecurity, eat unhealthy diets, and have higher levels of dietary-related diseases than are other groups [32]. People in the lowest socio-economic range are more likely than people at higher socio-economic levels to have to rely on processed foods for a larger portion of their diet, and thus are more likely to consume greater quantities of and experience the negative effects of trans fats on their health [14, 62, 78]. However, this is a paradigmatic example of how failures in the food supply that most affect the worst-off have effects on all members of a society. Everyone who eats processed foods will experience the negative health effects of trans fats to some degree. This includes the best-off in society. Removing trans fats from the food supply increases equity by protecting the health of those who must rely on processed foods more often, while promoting the health of everyone, and improving the overall health of the entire community. Given this, bans on trans-fats in foods have been introduced in numerous jurisdictions [29, 31, 39]. This is an example of a population-level regulatory change, not an individual-level change, which improves the health of everyone, but especially those who are worse-off in society. Approaching trans fats via regulatory change is sensitive to the social determinants of health, and is a more comprehensive approach to this particular food ingredient, than ‘nudging’ individuals to stop choosing processed foods.

With regards to sugar, Public Health England (PHE) has recently published guidelines for the food industry restricting the amount of sugar added to food in the United Kingdom [58]. The food categories covered by the guidelines include the likely suspects, such as ice cream and cake which one would expect to contain sugar, but also some possibly surprising candidates such as breakfast cereals and yoghurts [58]. In North America, the Centre for Science in the Public Interest reports that sugar is added to upwards of 80% of processed foods [43]. Additionally, there is now some evidence that sugar has negative impact on the body’s metabolic functioning [58]. Given that processed foods covered by a trans fats ban also contain high levels of sugar, and that the social groups more likely to rely on processed foods face the brunt of the corresponding negative health impacts, regulations on sugar similar to those regarding trans fats may be called for, and may have similar equity-improving effects. The high levels of sodium currently added to processed and restaurant foods represents yet another area where regulation would be appropriate, and would have important health and equity effects [43, 48].

This is not to say that insights from the behavioural sciences have no place in public health policy. Consider, for example, Warin et al.’s report on empirical work in South Australia. The team of researchers found that individuals of low socio-economic status experience ‘shortened horizons’, and do not conceptualise health or ‘the future’ in the way that public health and policy-makers typically do [77]. This group of people focus their mental, physical, and financial resources on coping with present needs, so the notions of protecting health for a future time, or investing in one’s health, are less present in their lived experiences. Thus, most forward-looking public health messages are not effective on or sensitive to this group of people [77]. This research has interesting implications for the use of interventions based on behavioural science. In particular, it suggests that nudges in the context of immediate choices could be more effective interventions than education or social marketing for *certain groups* in *certain environments*. This is not (just) because they manipulate sub-consciously, but because they meet this group of decision-makers within their operative and prioritised set of concerns. Having said that, this application of nudges does not address the deeper problems that this group faces, in terms of employment, income, housing, education, or other social determinants. In this regard, regulatory changes, such as banning trans fats from processed foods, or limiting the amount of additive sugars, promote the health of the population without requiring that individuals in this group (or any group) actively commit to changing their behaviour, thus avoiding extra psychological burdens. What this shows is that there is a range of daily life in which behavioural science can help us to address certain proximal influences on health, but it must work in cooperation with regulatory measures that address the distal social determinants of health.

In advocating these regulatory and population-level measures, an important question arises regarding the role and function of public health (policy). Our argument below is that an equitable and justifiable public health aims at more than individualised interventions targeting the usual public health suspects.

## (In)equality and Social (In)justice II: What Kind of Public Health?

A common interpretation of public health is that it is a branch of the state that acts to protect communities from infectious diseases. This focus can justify interventions that restrict individual liberty based on the risk of harm to others, and permit the legitimate use of various state powers, including quarantine, in order to limit communicable disease outbreaks [21, 55]. This way of conceptualising public health may work well for infectious disease or cases (like smoking) where collateral harm to others from the individual’s choice can be clearly established. In order to protect other people from harms, public health may exercise state powers and interfere with individual liberty with some level of legitimacy. This is typically the case in public health measures focused on communicable disease. However, this way of conceptualising public health poses challenges when public health is called upon to intervene to address non-communicable diseases. In cases of non-communicable disease, as well as diseases where there is not a clear link of collateral harms to others, public health interventions that involve some restriction upon people’s liberty (even when small) may not be as easy to justify on this model of public health action. Since people’s failures to undertake public health measures regarding, for example, food choices do not clearly put the health of other people at risk, public health may be limited in the kinds of ‘liberty-restricting’ policies that they may (legitimately) introduce.

However, what counts as restricting liberty in an unjustified manner is a matter of perspective and debate. On some liberal views, there seems to be a low tolerance for measures like governing portion sizes of foods, or penalties (in the form of taxes). On such views many public health initiatives have been labelled ‘nanny’ interventions (often after the lobbying and media efforts of food companies). Here, ‘traditional’ public health interventions, such as those addressing sanitation or viral outbreaks, are seen as more legitimate spheres of action for the state. Communicable diseases are viewed either as potential harms to others or as failures of important community systems/infrastructure (that could provide protection from such diseases) [52, 65]. Interventions to prevent these can be contrasted with public health interventions regarding the ‘usual suspects’. These are perceived, on such views, as addressing *individual* choice as opposed to communicable or other disease vectors. Where public health is framed in this manner, there is a justificatory dilemma. People’s failures to undertake public health measures addressing things like dietary choices may not justify public health interventions that restrict people’s liberty [1, 49, 65]. Yet, focusing too much on individual liberty may actually have a negative impact on the health of the public, and so should not be taken as granted [20].

What is often missing from more individual liberty-focused accounts of public health is the recognition that some non-communicable diseases are causally connected to the SDoH. As such, there are a range of causal factors which are largely out of the individual’s control [34]. As mentioned previously, there is wide recognition and agreement that the social determinants of health, including education, employment, housing, and food security, all contribute to a person’s health state [68]. A person’s ability to make individual health-protective choices is determined to a great degree by these factors, and public health interventions that do not attend to this may miss their target group or inadvertently deepen inequities [17, 77].

An alternative and, some have argued, more appropriate perspective views public health’s purpose as achieving and supporting the necessary conditions for justice and health equity [49, 55]. This interpretation permits a focus on the social determinants, and highlights the need for regulatory change in order to address many contributors to poor health. Attention to this is not necessarily at odds with the tactics of behavioural science, but is a crucial counterpart to any nudge-based intervention in choice architecture [10, 70]. A model of public health which places the social and other determinants of health at its centre would seek to address the distributional failures at the source of non-communicable disease. This would move the emphasis away from individual responsibility, as well as away from long-standing anxieties about personal liberty and worries about (unjustified) interference with individual choices about self-regarding health behaviours [1, 54]. It would concentrate on systemic and distributional problems, rather than making health issues the sole domain of the individual [55]. Given this, if the achievement of a base level of human well-being for the population is the goal of a system of public health, then the largely individual-focused approach of behavioural science-based public health policy as it currently stands may not be fit for purpose.

## Concluding Remarks: Mitigating Inequalities and Addressing Determinants

In the extensive and ever-burgeoning literature on behavioural science, libertarian paternalism, and nudges, very little seems to engage directly with the SDoH. Although commentators discuss the appropriateness of nudges and behavioural science to address individual behaviour change (on ethical, legal, or policy terms), the social determinants of health are often only mentioned in passing. There is a real and present danger that the influence of behavioural science and libertarian paternalism in recent years has undermined the earlier consensus on the need for an ‘ecological’ approach to health. The benefit of this approach was that it placed the individual factors involved in ill health behind the structural and socially-determined factors. Solutions addressing the social factors would have perhaps gained more attention if this model had maintained or gained influence. Instead considerations of the way that findings in behavioural science do or do not intersect with knowledge about the social determinants of health seem to have been largely neglected.

At least on the face of it, the intellectual approach in the behavioural sciences represents a welcome change from the questionable assumptions about rationality and decision-making which have been embedded in more traditional approaches to law and policy. By focusing on the contextual factors affecting decision-making, they hold the potential to increase the effectiveness of policy and regulatory interventions. That said, there is a great need not to allow our policy-makers to become blinded by nudge-type interventions addressing the proximal influences on health, at the expense of the many distal influences. Addressing the social determinants is essential to making any long-term progress on ill-health, specifically when considering certain non-communicable diseases. This seems especially relevant given the on-going concerns about the quality of evidence supporting certain behavioural science interventions, as even behavioural scientists themselves admit [66]. We ought to bring the application of behavioural science closer to the idea that seems part of its origin: that behavioural science may help regulation work better, but cannot replace regulation entirely [72]. In theory, experimentation in behavioural science could be improved by using groups that are more representative and diverse, or at least more similar to the target group for a given intervention, and by broadening the range of what counts as good evidence. Behavioural science could, in theory, also be used in a way that supplements regulation which tackles systemic issues, or takes account of the social determinants of health, or helps to tackle the root causes of ill-health and health inequalities. Of course, whether these happen in practice is a matter of ethics, of continuing to emphasise the importance of systemic issues to everyone’s health, and of political will.

## Footnotes

1 We recognise that the behavioural sciences do and ought to include much more than this (e.g. the behavioural-focused social sciences), but often when the term is used in the types of policies and literature we refer to, it is used to mean these behavioural economics and cognitive psychology. Thus our intention in following this is not to create a behavioural straw-person argument, but to make clear that the target of our critique is a particular framing of the research and issues.

2 However, note that some of this is contested. As we will see in “(In)equality and Social (In)justice I: Methodological and Empirical Concerns” section, Gigerenzer demonstrates that proponents of these kinds of approaches to law and policy take a selective reading of the evidence.

3 Recently, the BIT has claimed regulations as ‘behavioural’, when, as we argue later in the paper, they are in fact not behavioural, but rather, old-fashioned legislative measures. For example, the UK is considering implementing a sugar-reduction strategy in April of 2018 that would place soft drinks (among other products) into different tax categories depending added sugar content. The goal of the policy seems to be to have companies change the formulation of their products, to lower the amount of sugar people consume *without* changing consumers' behaviour. We argue that this is not a behavioural intervention, but regulation *simpliciter*, with higher taxation as a penalty to noncompliant companies. This piece of old-fashioned regulation appears to be working, and we have critiqued behavioural scientists for claiming too much for themselves by framing this as a behavioural intervention. We briefly say more about this regulation in “Individual Incremental Gains or Across the Board Regulation?” section, below. Also see [36].

4 A literature review was conducted to discover the extent to which behavioural science-inspired/nudge-type interventions were being discussed, recommended, or critiqued in connection with the social determinants of health. A search was conducted using journal databases (JStor, SSRN, Google Scholar) and then within specific journal archives (Journal of Economic Psychology, Journal of Socio-Economics (now the Journal of Behavioural and Experimental Economics), Journal of Public Policy, Journal of Public Health, Public Health Ethics, Public Health Reports), using keyword combinations. The keywords on the right were matched with all of the keywords on the left in their possible combinations. The results from these searches were combed for articles that contained both sets of keywords, and that addressed the social determinants of health (SDoH) as a central feature of the article. These keyword combinations were:

5 Such ideological and practical influences “are not insignificant and currently include a resurgence of… positive reinforcement and/or suggestion to influence behaviour” (p. 159).

6 Though these problems may be avoided as interventions develop. See Ref. [66].
